# Integration of individual prediction index based on autophagy‐related genes and clinical phenomes in melanoma patients

**DOI:** 10.1002/ctm2.132

**Published:** 2020-08-15

**Authors:** Ming Ren, Chuan‐Yuan Wei, Lu Wang, Xin‐Yi Deng, Nan‐Hang Lu, Jian‐Ying Gu

**Affiliations:** ^1^ Department of Plastic Surgery, Zhongshan Hospital Fudan University Shanghai China; ^2^ Department of Liver Surgery, Liver Cancer Institute, Zhongshan Hospital, Key Laboratory of Carcinogenesis and Cancer Invasion, Ministry of Education Fudan University Shanghai China

Dear Editor,

High metastasis and mortality are characteristics of melanoma,^1^ and it is a serious threat to human health. Autophagy plays an important role in melanoma progression,^2^ and therefore, the aim of our study is to investigate valuable biomarkers related to autophagy, moreover, to construct individual prediction index instead of “one size fits all” index for melanoma.

**FIGURE 1:**
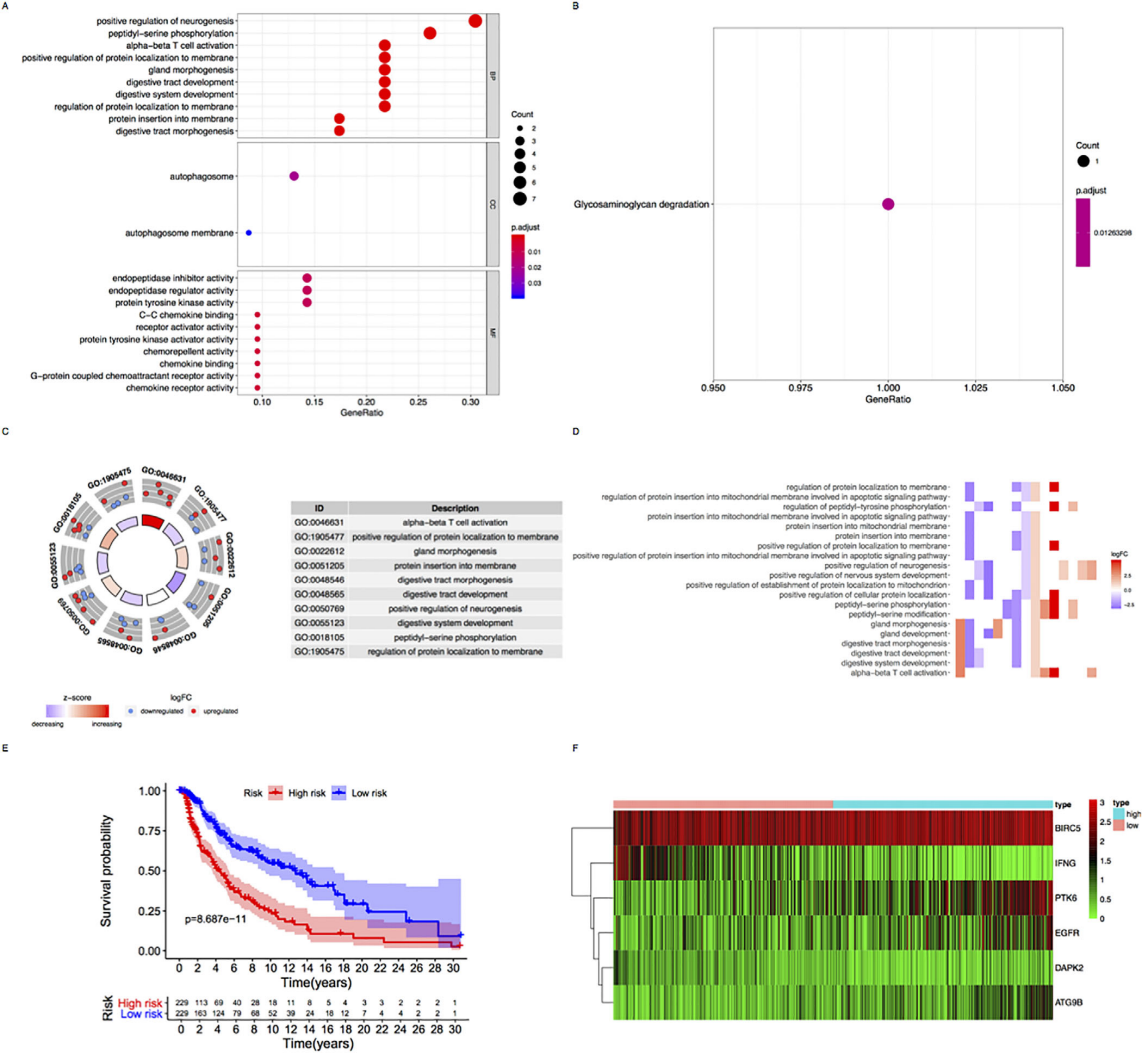
Identification and functional analyses of autophagy‐related genes. A, GO bubble plot: The *y*‐axis is the function, and the *x*‐axis is the proportion of genes corresponding to the function in the total differential genes. The color scale represents the significant difference. B, KEGG bubble plot: The most significant pathway for the enrichment is glycosaminoglycan degradation. C, Circle plot: The relationship between ARGs and corresponding path. The red dot is upregulation, and the blue dot is downregulation. D, A heatmap of the relationship between ARGs and pathways. E, Survival analysis between high‐risk group and low‐risk group. F, Heatmap of six key genes in TCGA. The red part is upregulation, and the green is downregulation

We downloaded data of 461 cutaneous melanoma samples including gene expression and clinical information from the Cancer Genome Atlas (TCGA) database (http://cancergenome.nih.gov/), and 471 normal skin samples from the Genetype Titanium Expression (GTEx) database (https://www.gtexportal.org/home/datasets). Then we screened and combined the gene data of significant expression between cutaneous melanoma and normal skin tissue by R and SPSS software (*P *< .05). We also downloaded autophagy‐related genes (ARGs) from the Human Autophagy Database (HADb) (http://www.autophagy.lu/index.html), extracting the expression of ARGs from the combined matrix. There were 23 genes that met the screening criteria |log2 (fold change)| > 1 and adjust *P* < .05, and we found 14 upregulated genes (IFNG, FAM215A, NKX23, PRKCQ, NRG3, CCR2, NLRC4, IL24, CXCR4, BIRC5, BCL2, ARSB, SERPINA1, and APOL1) and nine downregulated genes (DAPK2, PTK6, NRG1, TP63, ATG9B, EGFR, ATG16L2, SPNS1, and TP73) (Figure 3A‐C). Then, we used Gene Ontology (GO) and Kyoto Encyclopedia of Genes and Genomes (KEGG) to analyze (Figure 3D), and the function of glycosaminoglycan degradation was mainly enriched, which reflected that it was the key process in autophagy metabolism. Meanwhile, the results also showed the most significant KEGG pathway was decreased, so we speculated that autophagy has complex regulation in tumor, though, on the whole, it suppressed melanoma progression (Figures 1A‐D).

We used univariate and multivariate Cox regression model (*P *< .05) to screen out six genes (IFNG, DAPK2, ATG9B, PTK6, BIRC5, and EGFR) that related to overall survival (OS) of patients (Figure 3E). As some studies have proved that, IFNG induces PD‐L1, expressed on the surface of melanoma cells, to avoid immune surveillance.^3^ In v‐raf murine sarcoma viral oncogene homolog B1 (BRAF) mutant melanoma, mitogen‐activated protein kinase (MAPK) signaling pathway will be amplified, consequently, the phosphorylation of ERK will directly activate DAPK2, leading to mitochondrial fusion; finally, the signaling pathway that accelerates tumor angiogenesis will be upregulated.^4^ ATG9B protein is involved in the formation and transportation of autophagosome, and the overexpression of ATG9B is related to the deterioration of some cancers.^5^ In addition, PTK6 can activate the pathway of EGFR, and then EGFR triggers a series of signal transduction. Two main downstream signal pathways are phosphatidylinositol 3‐kinase (PI3K)/protein kinase B (Akt)/phosphatase and tensin homolog deleted on chromosome ten (PTEN)/mammalian target of rapamycin (mTOR) and Ras/Raf/extracellular signal regulated kinase (MEK)/extracellular regulated protein kinase (ERK), which will inhibit cell apoptosis and promote tumor growth.^6^ BIRC5 encodes survivin, which has been found that high expression of survivin is closely related to cancer progress and drug resistance.^7^ More importantly, some inhibitors are currently being designed to block the progression of cancer mediated by BIRC5, EGFR, and so on.^6^, ^8^ It suggests that these biomarkers have great potential as therapeutic targets for melanoma patients.

**FIGURE 2:**
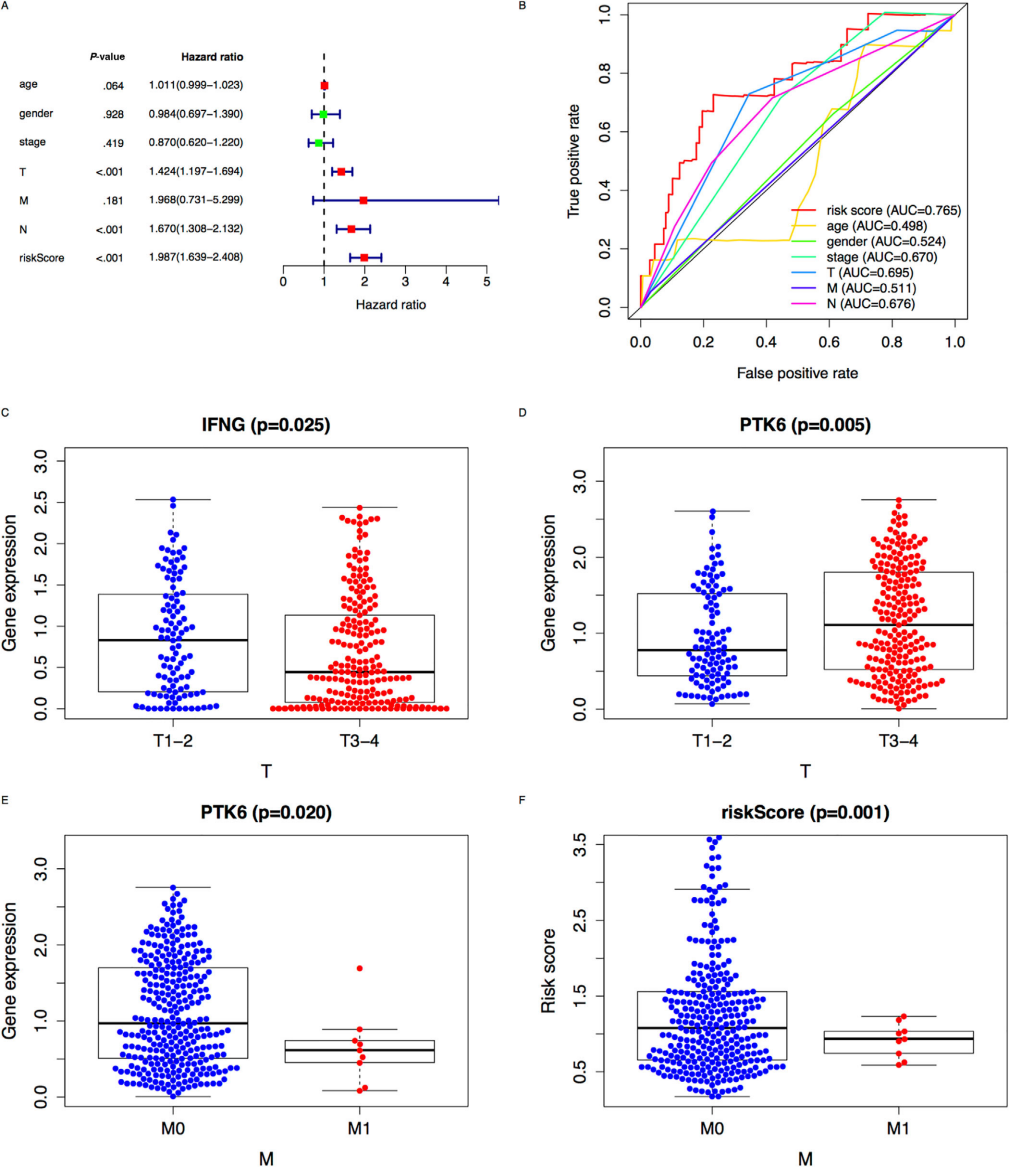
Validation of prediction model and correlation between ARGs and clinical parameters. A, Multiple regression analysis of clinical parameters and PI. B, Time‐dependent receiver operating characteristic (ROC) curve analysis of clinical parameters, and AUC value represented the prediction performance of each parameter. C, Expression of IFNG in T stage melanoma. D, Expression of PTK6 in T stage melanoma. E, Expression of PTK6 in M stage melanoma. F, Distribution of risk score in M stage melanoma

**FIGURE 3:**
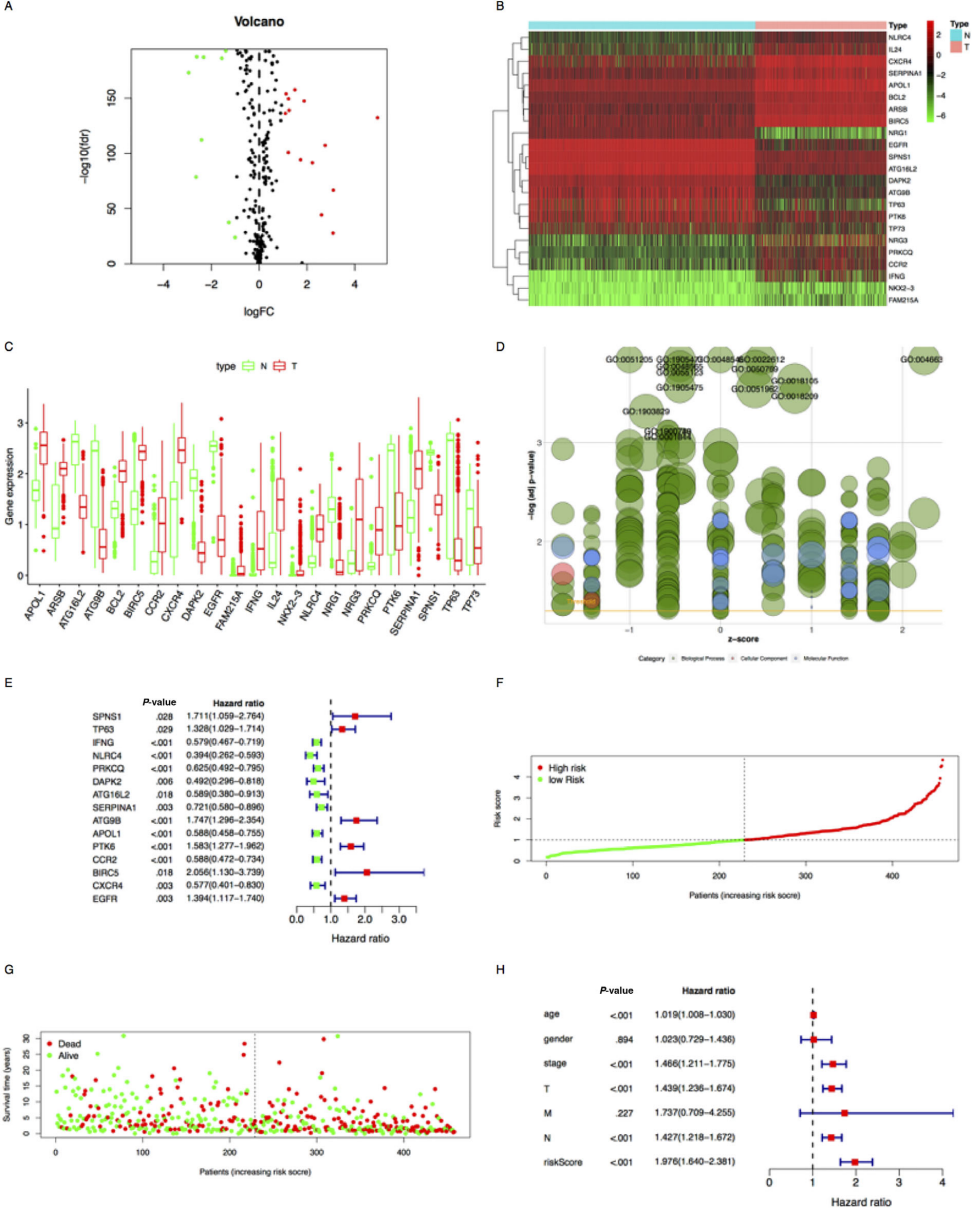
A, Volcano plot: In the volcano plot of differential gene expression, red dot indicated high expression, green dot indicated low expression, FDR was false discovery rate, and logFC was log(foldchang). B, Heat map: The ordinate represents gene clustering and the color scale represented gene expression abundance—the red part was upregulation and the green was downregulation. C, Box plot: Expression of 23 ARGs in melanoma and normal skin tissues. The red part was the gene expression of tumor samples, green was normal skin tissue, and each scattered dot represented a sample. D, GO bubble plot: Each circle represented a function corresponding to the related gene. The height of the circle represented a statistically significant difference. The size of the circle represented the proportion of differential genes in the corresponding function. Green was biological process (BP), red was cell composition (CC), and blue was molecular function (MF). E, Univariate Cox regression analysis of ARGs and patient OS. F, PI distribution of patients in TCGA. G, Survival status of patients in TCGA. H, Univariate Cox regression analysis of clinical parameters and PI

We constructed prediction index (PI) with these genes, and the formula was that the level of gene expression multiplied by regression coefficient, as PI = [expr(IFNG) × (–0.39)] + [expr(DAPK2) × (–0.66)] + [expr(ATG9B) × (0.33)] + [expr(PTK6) × (0.41)] + [expr(BIRC5) × (0.77)] + [expr(EGFR) × (0.36)]. Then we took the median value of PI to distinguish the high‐risk group from the low‐risk group (Figure 3F‐G), and the result showed that OS in low risk group was higher than that in high risk group by K‐M survival analysis (*P *< .05) (Figure 1E). We also drew heatmap about the six genes (Figure 1F). These findings showed that maintaining low expression of IFNG (Coef = –0.386, *P* = .002) and DAPK2 (Coef = –0.662, *P* = .022) was an effective protective factor that could prolong OS in melanoma patients, whereas ATG9B (Coef = 0.332, *P* = .038), PTK6 (Coef = 0.411, *P* = .000), BIRC5 (Coef = 0.77, *P* = .014), and EGFR (Coef = .357, *P* = .004) could reduce OS and accelerate the progression of melanoma. In order to verify the predictive ability of the model, we used the clinical parameters (from TCGA) and PI of these patients to make univariate and multivariate Cox regression analyses (*P *< .005) (Figure 2A, 3H), and then we drew time‐dependent receiver operating characteristic curves and calculated the area under the curve (AUC) (Figure 2B). We found PI was the most predictive factor (AUC = .765) among all clinical parameters, followed by T stage (AUC = .695) and N stage (AUC = .676). Furthermore, we conducted Cox regression analyses to reveal the correlation among ARGs, PI, and clinical parameters. Interestingly, in T3‐4 stage of melanoma patients, IFNG (*P* = .025) and PTK6 (*P* = .005) were highly expressed, which indicated that IFNG and PTK6 promoted tumor growth and invasion (Figures 2C‐D). However, PTK6 in M0 was significantly higher than that in M1 (*P* = .002) (Figure 2E), which demonstrated that PTK6 made tumor get worse, but not in the process of metastasis. Additionally, PI was closely related to M0 (*P* = .001) (Figure 2F), which showed PI had better predictive ability especially in patients without metastasis.

In our research, autophagy plays an important role in the inhibition of melanoma progression. Moreover, we identified that these ARGs (IFNG, DAPK2, ATG9B, PTK6, BIRC5, and EGFR) were significantly correlated to the OS of melanoma. Most of the molecules are being explored as new targets for tumor treatment, and it demonstrates that the six ARGs are potential and valuable biomarkers for prognosis and treatment of melanoma. Besides, because PI was based on individual level of gene expression, PI showed a good predictive performance, especially in melanoma without metastasis. However, the limitation of this study lies in its retrospective nature. Our results need to be verified in prospective clinical trials, and we need to make further efforts to reveal the significance of these biomarkers in future studies.
